# Long term dental transversal stability of Class II division 1 treated with cervical headgear

**DOI:** 10.1590/2177-6709.27.2.e2220291.oar

**Published:** 2022-06-10

**Authors:** Márcio Bastos de OLIVEIRA, Jean Nunes dos SANTOS, Vanessa Mascarenhas LIMA, Tiago Fonseca Lima da FONTE, Telma Martins de ARAUJO, Carlos Jorge VOGEL, Emanuel Braga RÊGO

**Affiliations:** 1 Universidade Federal da Bahia, Faculdade de Odontologia, Pós-graduação em Odontologia e Saúde (Salvador/BH, Brazil).; 2 Universidade Federal da Bahia, Faculdade de Odontologia, Departamento de Ortodontia (Salvador/BH, Brazil).

**Keywords:** Relapse, Angle Class II malocclusion, Orthodontic treatment, Stability

## Abstract

**Introduction:**

In several conditions, outcome stability is a great challenge for Orthodontics. Previous studies have reported that relapse commonly occurs along the years after orthodontic treatment finishing.

**Objective:**

The aim of the present study was to evaluate in the long-term transversal dental arch changes of Class II division 1 patients treated with cervical headgear and fixed appliance.

**Methods:**

Plaster study casts of 20 patients treated with cervical headgear without dental extractions were 3D-scanned and evaluated in three distinct times: initial (T_1_), immediate post-treatment (T_2_) and long-term retention (T_3_ - minimum 20 years). Transversal teeth distance of maxillary and mandibular canines, premolars and first molars were measured.

**Results:**

A statistically significant increase during treatment was observed for all maxillary teeth transversal distances (*p*< 0.05). In turn, a significant reduction was observed in the long term (*p*<* *0.05). For the mandibular teeth, canine transversal distance presented statistically significant constriction in the retention period (*p*<* *0.05). Mandibular first molars distance was significantly expanded by treatment (*p*<* *0.05) and remained stable in the long term. The changes observed for the other teeth or other times were considered not statistically relevant.

**Conclusions:**

For the accessed sample, transversal changes occurred during treatment and retention phases in Class II division 1 patients treated with cervical headgear and fixed appliance. Relapse was considered statistically relevant, even with the institution of a retention protocol.

## RESUMO

**Introdução:**

Em várias condições, a estabilidade dos resultados é um grande desafio para a Ortodontia. Estudos prévios relataram que a recidiva ocorre, comumente, ao longo dos anos, após o término do tratamento ortodôntico.

**Objetivo:**

O objetivo da presente pesquisa foi avaliar as alterações transversais da arcada dentária em longo prazo de pacientes Classe II, divisão 1, tratados com aparelho extrabucal cervical e aparelho fixo.

**Métodos:**

Modelos de gesso de 20 pacientes tratados com AEB cervical, sem extrações dentárias, foram escaneados e avaliados em três momentos distintos: inicial (T_1_), pós-tratamento imediato (T_2_) e acompanhamento de longo prazo (T_3_, mínimo de 20 anos). A distância transversal entre os caninos superiores e inferiores, pré-molares e primeiros molares foi medida.

**Resultados:**

Foi observado aumento estatisticamente significativo durante o tratamento para todas as distâncias transversais dos dentes superiores (*p* < 0,05). Por sua vez, foi observada redução significativa em longo prazo (*p* < 0,05). Para os dentes inferiores, a distância transversal intercaninos apresentou constrição estatisticamente significativa no período de contenção (*p* < 0,05). A distância dos primeiros molares inferiores aumentou significativamente com o tratamento (*p* < 0,05) e permaneceu estável em longo prazo. As alterações observadas para os outros dentes ou outros tempos foram consideradas sem significância estatística.

**Conclusões:**

Para a amostra estudada, as alterações transversais ocorreram durante as fases de tratamento e contenção em pacientes Classe II, divisão 1, tratados com aparelho extrabucal de tração cervical e aparelho fixo. A recidiva foi considerada estatisticamente significativa, mesmo com a instituição de um protocolo de contenção.

## INTRODUCTION

Orthodontic treatment aims at achieving adequate functional and aesthetics aspects of the dental and maxillofacial complex, thus promoting better life quality. Treatment outcome stability is of great interest for both professionals and patients; however, it is still considered a challenge. Transversal dental changes are commonly observed after appliance removal and several studies have shown progressive stability loss.^1^ ^-^ ^7^ In the other hand, the literature also provides evidences of balance, usually presenting reduced width modifications over time.^8^ ^,^ ^9^

Class II division 1 patients frequently present significant constriction of maxillary dental arch.^10^ Studies aiming at evaluating Class II patients during active growth stage treated without dental extraction have noted that during treatment molar area is significantly expanded, remaining stable in the retention period.^3^ ^,^ ^9^ However, the great majority of the studies evaluated short retention periods.^3^ ^,^ ^5^ ^,^ ^8^ ^,^ ^11^ ^,^ ^13^ ^-^ ^18^ Moreover, these researches were mainly performed using post-graduation programs sample, a design in which patients are treated by a varied sort of techniques or professionals and with several retention protocols. 

Headgear therapy has proved to effectively assist on managing Class II malocclusion in growing patients. Classical articles,^19^ ^,^ ^20^ more recent researches^21^ ^-^ ^23^ and updated meta-analysis^24^ have demonstrated positive skeletal effects with the use of extraoral forces applied to the maxillary bone. However, occlusal stability is not well addressed in the Class II treatment studies. Understanding teeth behavior in the retention phase is considered crucial for good professional practice and patient expectations fulfillment. A relatively recent survey has found that despite a decline trend in the use of headgear in USA/Canada, the majority (62%) of the interviewed practitioners were still using the device for Class II correction.^25^

In this context, the present study aimed at evaluating long term transversal changes (mean period of 25 years retention) using a sample of Class II division 1 patients treated with cervical headgear and no extractions, conducted by a single experienced operator employing the same technique and similar retention protocols. 

## MATERIAL AND METHODS

The present study was performed using non-probability sampling method (convenience sample). To collect the sample, an experienced clinician actively sought former patients who had been treated from the mid 1970s to the early 1990s with the following initial diagnose criteria: (1) Angle Class II division 1 malocclusion with bilateral full Class II molar relationship; (2) vertical skeletal pattern within a normal range (FMA = 25?5?), (3) active growth potential, (4) no congenital agenesis and (5) no craniofacial anomalies or syndromes. Treatment employed in those patients comprised: (1) non-extraction (except third molars); (2) cervical pull headgear (500gf, 12h/day), 2mm laterally expanded in combination with 0.022 x 0.028-in Edgewise standard fixed appliance with no tip or torque in the brackets; and (3) absence of Class II intermaxillary elastics use. 

Patient’s records should present good quality lateral cephalograms and centric occlusion plaster study casts obtained at pretreatment (T_1_) and immediate post-treatment (T_2_). Finally, the following additional criteria were also verified for including the patient in the sample: (1) fulfillment of molar key occlusion in T_2_ (defined by the accurate occlusion of the mesiobuccal cusp of the maxillary first permanent molar in the groove between the mesial and the middle cusps of the mandibular first permanent molar); and (2) minimum of 20 years of treatment completion. 

From March 2012 to December 2016, a tireless attempt to make contact with patients attending the inclusion criteria was performed. From those who accepted to participate in the study, written informed consent was obtained and a lateral cephalogram and study dental casts were taken at the time of the recall appointment (T_3_). In this stage, patients could not present any tooth loss or major dental rehabilitations. Patients should not present dental anatomy deviations, agenesis or prosthetic rehabilitations. Treatment employing interproximal reduction, excessive tooth rotation in T_1_ and excessive cusp tip abrasion in T_3_ were excluded. 

A set of three dental casts was thus organized: initial, taken before any treatment (T_1_); post-treatment casts (T_2_); and retention casts, taken in the long-term recall (T_3_). 

**Figure 1:**
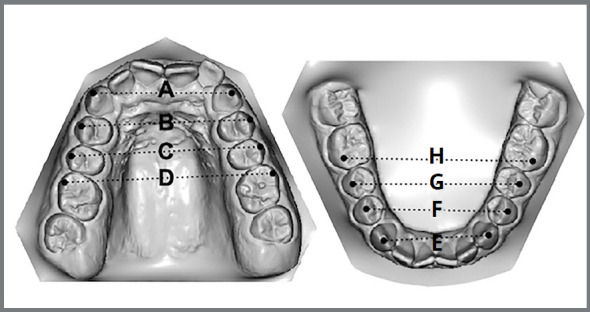
Transversal dental distances: **A**) maxillary inter-canines; **B**) maxillary inter-first premolars; **C**) maxillary inter-second premolars; **D**) maxillary inter-molars; **E**) mandibular inter-canines; **F**) mandibular inter-first premolars; **G**) mandibular inter-second premolars; **H**) mandibular inter-molars.

Dental casts of all periods were digitized using the Ortho Insight 3D scanner (LLC, Chattanoga, Tennessee, USA) and evaluated with the software Motionview (LLC, Chattanoga, Tennessee, USA). Measurements were automatically given by the software after cusp tip determination. For transversal measurement of canine, cusp tip was utilized as reference. For premolars and molars, vestibular and mesiobuccal cusp tip were used, respectively (Fig 1).

This longitudinal retrospective study was approved by the independent Ethics Committee of Federal University of Bahia, Dental School (n. 1.969.204).

### METHOD ERROR

Previously, aiming at determining examiner calibration, 5 patients were randomly selected using an online randomization program (*https://www.random.org/*). Same measurements were digitally obtained in two different periods with two weeks interval, under the same operational conditions. Reproducibility was evaluated using Pearson correlation coefficient, with 95% confidence level. Results have shown indexes greater than 0.97, thus indicating strong correlation among the measurements. 

### STATISTICAL ANALYSIS

Data were compiled and analyzed with SAS v. 7.1 software(SAS Institute Inc., Cary, NC, USA). For sample distribution, symmetry and kurtosis an examination was employed and revealed normal distribution of the data. Results have demonstrated no significance among sample size in the successive times of measurements and normality of data distribution. The comparison of the results measured in the different times was evaluated with paired *t*-test, using 95% as confidence level. 

## RESULTS

The search revealed 54 orthodontic cases meeting the inclusion criteria; 34 patients could not be found or refused to participate. Those who did not accept, reported living too far away, had scheduling conflicts, expressed radiation fears or simply refused to participate for unspecified reasons. Twenty patients (14 females and 6 males) agreed to attend the recall appointment and accepted to make part in the sample of the present research.

The mean period of headgear use was 2 years / 1 month, and the mean period of fixed appliance was 3 years / 3 months. Total treatment mean time was 4 years / 4 months. Table 1 shows the overall and individual characterization of the patients included in the study, by gender and age at the three phases and the total follow-up period. The mean ages in the evaluated phases were: T_1_ = 11 years / 9 months; T_2_ = 16 years / 4 months; T_3_= 43 years / 3 months. The overall long-term mean period in the recall appointment was 25 years. The protocol of retention and time of use are described in Table 2. 

**Table 1:** Characterization of patients included in the study by gender and age at pre-treatment (T1), post-treatment (T2), and long-term retention (T3) phases, and the follow-up period after the end of orthodontic treatment (T3-T2).

Patient	Gender	Age			Follow-up
		T1	T2	T3	T3-T2
1	F	11y 8m	15y 2m	41y 5m	26y 3m
2	F	10y 11m	14y 7m	36y 8m	22y 1m
3	M	12y	20y	52y 3m	32y 3m
4	F	11y 6m	16y 7m	44y 6m	27y 11m
5	F	10y 8m	15y 3m	35y 7m	20y 4m
6	F	13y 2m	18y 1m	46y	27y 11m
7	M	11y 5m	17y 9m	42y 6m	24y 9m
8	F	12y 1m	17y 2m	54y 5m	37y 3m
9	F	11y 1m	15y 9m	39y 5m	23y 8m
10	F	12y 9m	17y 4m	46y 2m	28y 1m
11	F	10y	14y 4m	44y 4m	30y
12	F	11y	14y 1m	41y 1m	27y
13	F	12y 1m	15y	41y 1m	29y 1m
14	M	10y	16y 1m	39y 2m	22y 6m
15	F	14y 6m	16y 1m	40y 1m	24y
16	M	12y 11m	17y 6m	40y	22y 6m
17	F	10y 3m	16y 1m	45y 4m	29y 3m
18	F	13y 2m	14y 7m	52y 5m	37y 8m
19	M	14y 7m	18y 7m	39y 2m	20y 5m
20	M	13y 5m	16y 7m	43y 7m	27y
Overall (mean)		11y 9m	16y 4m	43y 3m	25y

F = female; M = male. Follow-up period: y = years, m = months.

**Table 2:** Gender; age of patients at Pretreatment (T1), Immediate post-treatment (T2) and Long-term evaluation (T3); Total time of retention (TTR); Retention protocols used for the maxillary and mandibular arches.

Patient	Gender	T1	T2	T3	TTR	Maxillary Retention	Mandibular Retention
1	F	11.8	15.2	41.5	26.3	R(10m CU/ 10y NO)	F(10y) + R(2y NO)
2	F	10.11	14.7	36.8	22.1	R(6m NO)	R(6m NO)
3	M	12	20	52.3	32.3	R(10m CU/ 6m NO)	R(10m CU/ 6m NO)
4	F	11.6	16.7	44.6	27.11	R(1y CU/ 8y NO)	R(1y CU)
5	F	10.8	15.3	35.7	20.4	R(1y CU/ 1y NO)	R(1y CU/ 1y NO)
6	F	13.2	18.1	46	27.11	R(6m CU)	R(6m CU)
7	M	11.5	17.9	42.6	24.9	R(6mCU/ 6m NO)	R(6m CU/ 6m NO)
8	F	12.1	17.2	54.5	37.3	R(1y CU/ 1y NO)	R(1y CU/ 1y NO)
9	F	11.1	15.9	39.5	23.8	R(1y CU/ 1y NO)	F(23y 8m)
10	F	12.9	17.4	46.2	28.1	R(6m CU)	R(6m CU)
11	F	10	14.4	44.4	30	R(8m CU)	R(8m CU)
12	F	11	14.1	41.1	27	R(2y CU/ 7y NO)	R(2y CU/ 7y NO)
13	F	12.1	15	41.1	29.1	R(1y CU)	F(1y)
14	M	10	16.1	39.2	22.6	R(1y CU/ 7y NO)	R(1y CU/ 7y NO)
15	F	14.6	16.1	40.1	24	R(6m CU/ 2y NO)	R(6m CU/ 2y NO)
16	M	12.11	17.6	40	22.6	R(1y NO)	F(22y)
17	F	10.3	16.1	45.4	29.3	R(1y CU/ 1y NO)	R(1y CU/ 1y NO)
18	F	13.2	14.7	52.5	37.1	R(1y CU/ 1y NO)	R(1y CU/ 1y NO)
19	M	14.7	18.7	39.2	20.5	R(1y CU/ 1y NO)	R(1y CU/ 1y NO)
20	M	13.5	16.7	43.7	27	R(1y CU/ 1y NO)	R(1y CU/ 1y NO)

F = Female; M = Male, T1 = pre-treatment, T2 = Immediate post-treatment; T3 = Long-term evaluation, TTR = Total time of retention, R = Removable; F = Fixed; m = months; y = years; CU = continuous use; NO = night-only use.

Table 3 shows mean and standard deviation of each measurement and p-value between the tested periods. It can be noted a statistically significant increase during treatment for all maxillary teeth transversal distances, followed by a significant reduction in the long term *(p*<0.05). For the mandibular teeth, canine transversal distance presented statistically significant constriction in the retention period (*p*<0.05). Mandibular first molars distance was significantly expanded by treatment (*p*<0.005) and remained stable in the long term. The changes observed for the other teeth or other periods were considered not statistically relevant.

**Table 3:** Mean, Standard deviation (SD) and p-value for each measurement at Pre-treatment (T1), Immediate post-treatment (T2) and Long-term evaluation (T3).

Variable (mm)	T1		T2		T3		T1-T2	T2-T3
	Mean	SD	Mean	SD	Mean	SD	p-valor	p-valor
Maxillary								
Inter-canines	32.61	1.84	34.59	1.23	33.81	1.39	<0.0001*	0.003*
Inter-first premolars	39.56	2.37	42.39	1.49	41.55	0.38	<0.0001*	0.000*
Inter-second premolars	44.60	2.39	48.14	1.73	47.26	1.97	<0.0001*	0.001*
Inter-first molars	49.61	2.63	52.72	1.90	52.12	2.14	<00001*	0.005*
Mandibular								
Inter-canines	26.34	1.63	26.54	1.19	25.16	1.70	0.525	<0.0001*
Inter-first premolars	33.61	2.22	34.64	1.39	33.96	1.74	0.024*	0.022*
Inter-second premolars	39.08	2.16	40.61	1.75	39.65	1.89	0.001*	0.000*
Inter-first molars	44.00	2.49	45.50	2.26	45.30	2.60	0.002*	0.312

## DISCUSSION

Relapse evaluation in the long term has always been subject of interest among orthodontists and researchers. In this regard, patient’s records have been source of comparison throughout the periods of treatment for quantification and severity of the alterations. Commonly, X-rays,^2^ plaster models^4^ ^,^ ^6^ ^,^ ^9^ ^,^ ^18^ or both X-rays and models^17^ are employed. The present study evaluated transversal dental changes using plaster study casts of patients treated using the same technique and employing similar protocol of retention for all patients. It is believed that treatment uniformity can be valuable for stability evaluation. The literature accessed showed few studies with similar methodology.^6^ ^,^ ^17^ The great majority of the published studies used samples belonging to post-graduation programs treated by various professionals, possibly using different techniques.^1^ ^,^ ^2^ ^,^ ^3^ ^,^ ^9^ ^,^ ^11^ ^,^ ^15^ ^,^ ^16^ ^,^ ^18^ ^,^ ^26^

The sample of the present study was treated without extraction, using cervical headgear and fixed appliance. Mean age in T_1_ was 11 years and 9 months, similar to previous studies that reported between 10.1 and 13.2 years^3^ ^,^ ^11^ ^-^ ^16^ ^,^ ^26^ as mean age for headgear therapy start. Retention minimum period of 20 years was set for the current research, and a mean of 25 years retention was achieved. A previous report from Little et al.^26^ brought similar retention period (27,8 years); however, most of the studies present shorter retention periods.^3^ ^,^ ^11^ ^-^ ^15^ It is believed that long periods of retention can bring more consistent evidences about stability. 

The choice of retention protocol can vary according to orthodontist experience. Retention appliances can be removable and/or fixed. By the time the patients were treated, removable Hawley appliance was generally used for the maxillary arch and Hawley or intercanine fixed 0.7-mm stainless steel wire, for the mandibular arch. Hoybjerget et al.^27^ did not observe any statistic difference comparing three retention protocols: upper and lower Hawley; upper Hawley and lower intercanine bar; upper Essix and lower intercanine bar. It is worth noting that retention success depends on patient’s compliance. This study brings the detailed retention use based on patients report (Table 2). Other studies have reported varied types of retention appliance,^6^ ^,^ ^9^ but did not describe the effectively used protocol.^5^ ^,^ ^8^ ^,^ ^17^ ^,^ ^18^

Angle Class II division 1 malocclusion is usually featured by the transversal constriction of the maxillary arch because of its anterior displacement in relation with the mandible. Increased overjet and overbite can be commonly observed. Intense lingual crown torque for posterior mandibular teeth is also commonly noted as compensation. Studies comparing Class II and Class I malocclusion showed a significant constriction of maxillary arch in Class II subjects.^10^ ^,^ ^27^ In this context, it is expected that after treatment, the maxillary arch becomes broader, since posterior area is progressively divergent. Mandibular posterior teeth tend to a mild expansion due to torque correction. 

A statistic significant increase during treatment was observed for all maxillary teeth distances, followed by a significant reduction in the long term (*p*<0.05). Pancherz et al.,^9^ evaluating 32-years retention period, and did not find statistic differences for the canines. Molars behavior was similar to the observed in the present study. 

In the current research, mandibular canine position was not significantly modified by the treatment. It is believed that this care is considered of great importance for outcome stability.^28^ ^-^ ^30^ On the other hand, during the retention period, a significant constriction was observed. Many studies have reported similar results for mandibular canines, and this feature seems to be well consolidated in the literature.^1^ ^-^ ^9^ Treatment promoted relevant expansion of the mandibular molars, followed by stability in the retention phase. Dyer et al,^6^ Park et al^8^ and Pancherz et al^9^ found very similar results regarding this measurement. 

Regarding premolars, few studies have targeted at measuring this feature. Bishara et al^3^ observed an increase of maxillary inter-second premolars distance during treatment in Class II division 1 patients treated without extraction. Dyer et al^6^ found relevant decrease of this area in the retention period, but the study used extractions, making difficult the comparison with the current research. 

Advantages and disadvantages may be attributed to the headgear therapy. Headgear is a very versatile device, permitting a varied sort of adjustments to fit to the specific morphological and growth pattern of the patient. Additionally, the device does not represent a high cost for the treatment, and is considered not difficult to be installed by the professional and/or worn by the patient.^31^ However, the success of the therapy is highly dependent on patient compliance.^32^ In addition, there is an increasing concern of children and parents regarding social and psychological aspects, and many professionals have tried to experience more aesthetic/discrete options or non-compliance approaches.^31^ ^,^ ^33^

Limitations of the present study are important to be highlighted. The research is retrospective/longitudinal and might introduce selection bias (ex: Are the patients satisfied with treatment outcome in the long term more willing to collaborate in the study? Why was female’s agreement to participate in the study much greater than males?). To minimize this problem, extensive search for patients who met the inclusion criteria was performed. A considerable number was found, but 20 accepted to join the study. The bias and the power presented by non-probability samples are usually not possible to be measured; however, convenience in some retrospective long-term researches in health sciences are justified by the ease of research, ready availability and cost effectiveness. Other limitation is the lack of untreated Class II malocclusion control patients with similar ethnic background. Although untreated Class II control collections are available for use, the authors of the present study assumed that a proper comparison would not be possible. 

Finally, despite many features have changed in a statistic manner, the magnitude of relapse may have discrete clinical implications. In this context, complementary studies are necessary to improve the understanding of the clinical significance of those changes. 

## CONCLUSION

In the period of at least 20 years of retention, the following transversal changes occurred during treatment and retention phases in Class II division 1 patients treated with cervical headgear:

» A statistic significant increase during treatment was observed for all maxillary teeth transversal distances, followed by a significant reduction in the long-term.
» Mandibular canine transversal distance presented statistically significant constriction in the retention period.
» Mandibular inter-first molars distance was significantly expanded by treatment and remained stable in the long-term.
» The changes observed for the other teeth or other periods were considered not statistically relevant.
